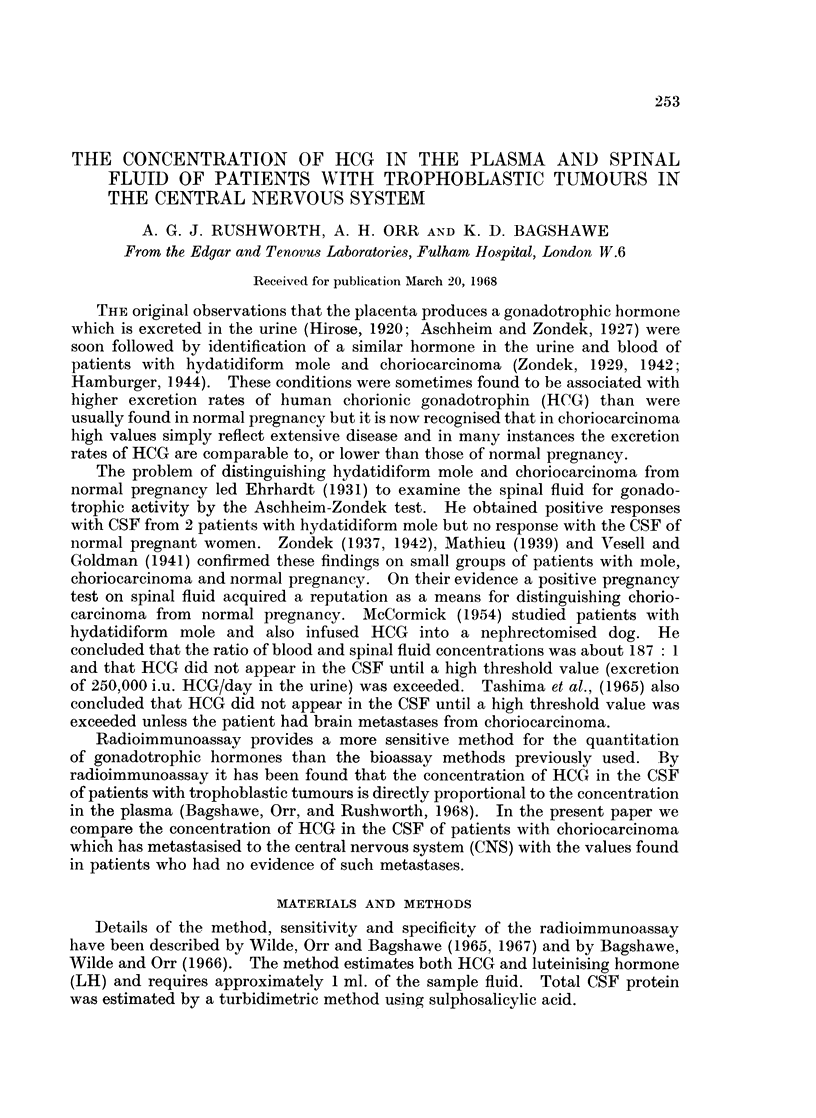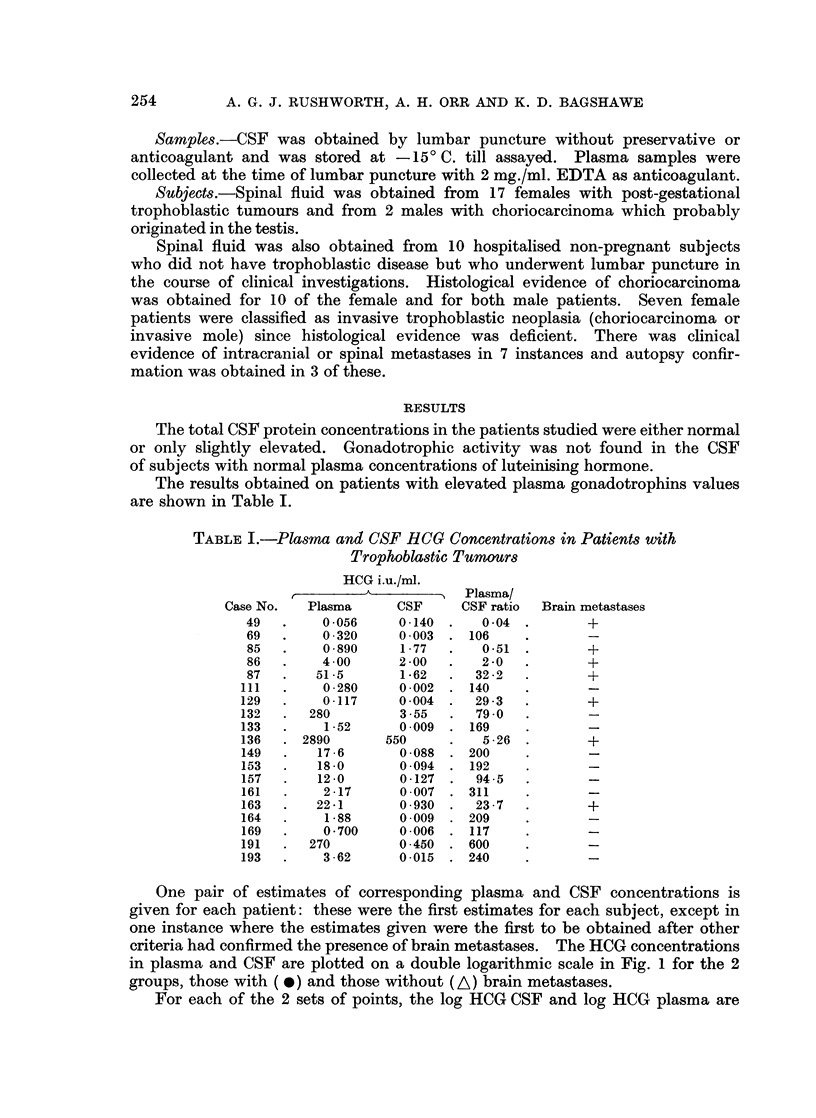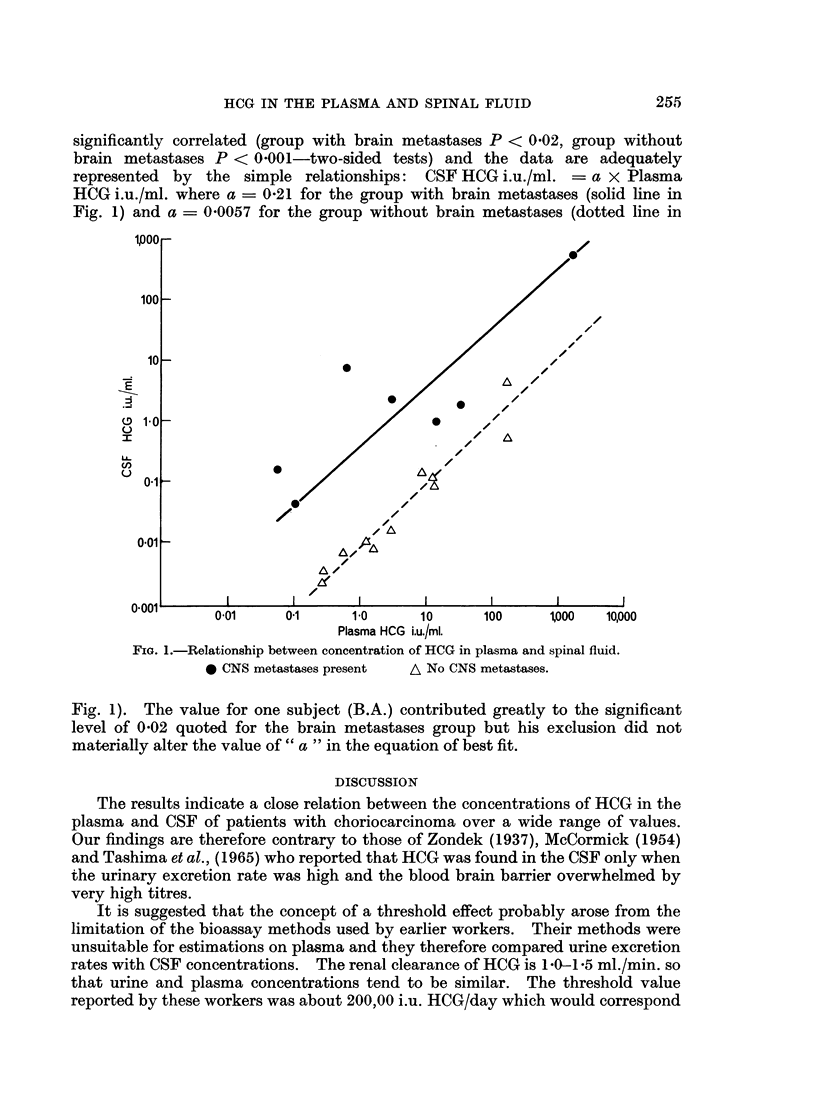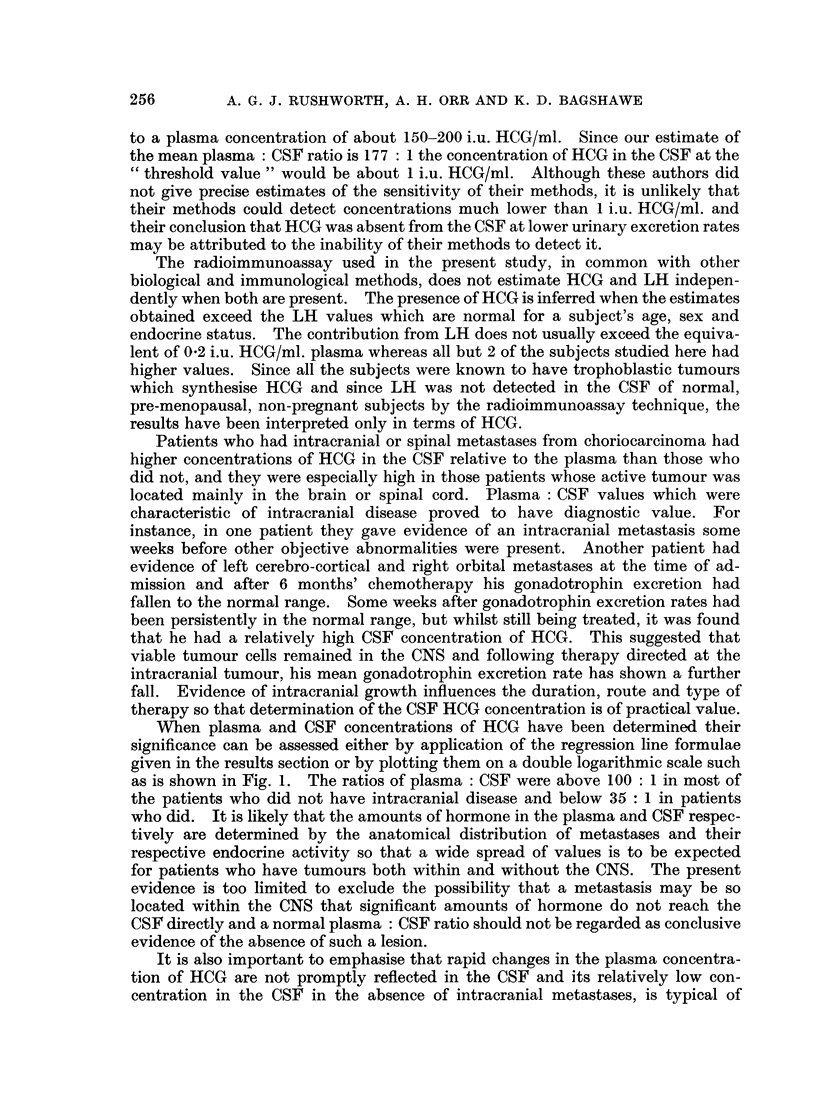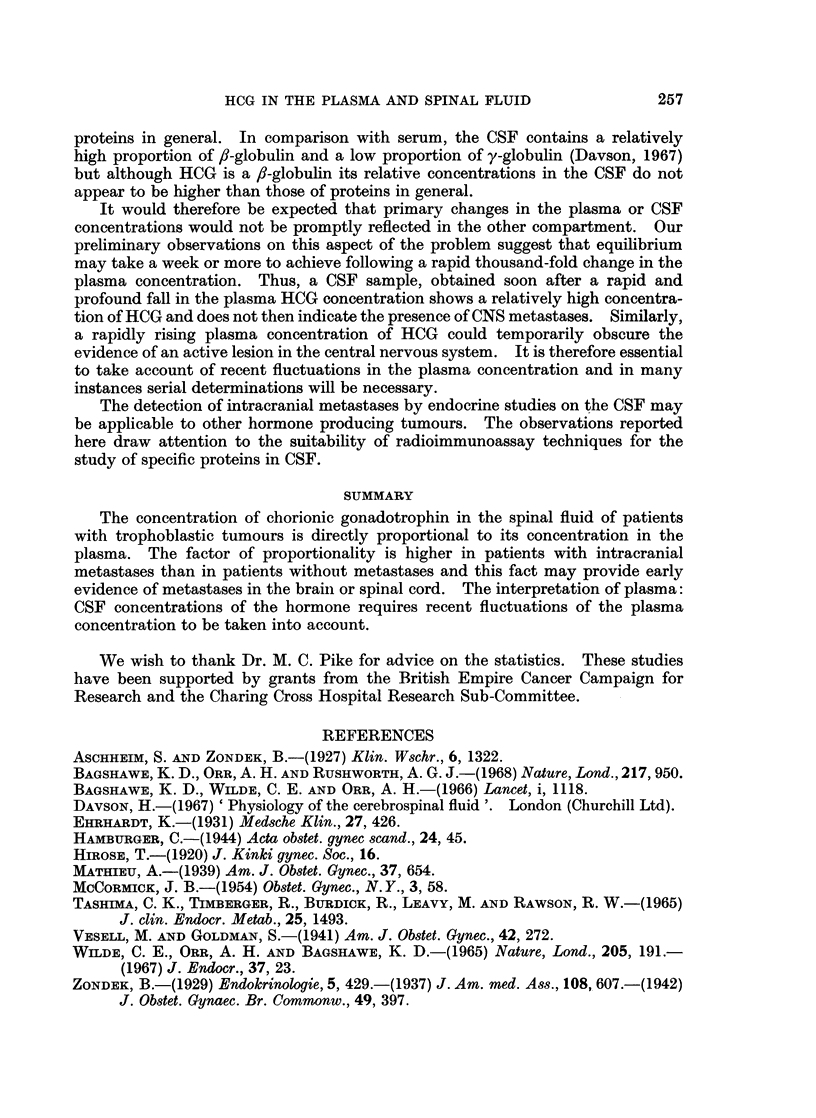# The concentration of HCG in the plasma and spinal fluid of patients with trophoblastic tumours in the central nervous system.

**DOI:** 10.1038/bjc.1968.33

**Published:** 1968-06

**Authors:** A. G. Rushworth, A. H. Orr, K. D. Bagshawe


### THE CONCENTRATION OF HCG IN THE PLASMA AND SPINAL FLUID OF PATIENTS WITH TROPHOBLASTIC TUMOURS IN THE CENTRAL NERVOUS SYSTEM

A. G. J. RUSHWORTH, A. H. ORR AND K. D. BAGSHAWE

*From the Edgar and Tenovus Laboratories, Fulham Hospital, London W.6*

Received for publication March 20, 1968

THE original observations that the placenta produces a gonadotrophic hormone which is excreted in the urine (Hirose, 1920; Aschheim and Zondek, 1927) were soon followed by identification of a similar hormone in the urine and blood of patients with hydatidiform mole and choriocarcinoma (Zondek, 1929, 1942; Hamburger, 1944). These conditions were sometimes found to be associated with higher excretion rates of human chorionic gonadotrophin (HCG) than were usually found in normal pregnancy but it is now recognised that in choriocarcinoma high values simply reflect extensive disease and in many instances the excretion rates of HCG are comparable to, or lower than those of normal pregnancy.

The problem of distinguishing hydatidiform mole and choriocarcinoma from normal pregnancy led Ehrhardt (1931) to examine the spinal fluid for gonadotrophic activity by the Aschheim-Zondek test. He obtained positive responses with CSF from 2 patients with hydatidiform mole but no response with the CSF of normal pregnant women. Zondek (1937, 1942), Mathieu (1939) and Vesell and Goldman (1941) confirmed these findings on small groups of patients with mole, choriocarcinoma and normal pregnancy. On their evidence a positive pregnancy test on spinal fluid acquired a reputation as a means for distinguishing choriocarcinoma from normal pregnancy. McCormick (1954) studied patients with hydatidiform mole and also infused HCG into a nephrectomised dog. He concluded that the ratio of blood and spinal fluid concentrations was about 187 : 1 and that HCG did not appear in the CSF until a high threshold value (excretion of 250,000 i.u. HCG/day in the urine) was exceeded. Tashima *et al.*, (1965) also concluded that HCG did not appear in the CSF until a high threshold value was exceeded unless the patient had brain metastases from choriocarcinoma.

Radioimmunoassay provides a more sensitive method for the quantitation of gonadotrophic hormones than the bioassay methods previously used. By radioimmunoassay it has been found that the concentration of HCG in the CSF of patients with trophoblastic tumours is directly proportional to the concentration in the plasma (Bagshawe, Orr, and Rushworth, 1968). In the present paper we compare the concentration of HCG in the CSF of patients with choriocarcinoma which has metastasised to the central nervous system (CNS) with the values found in patients who had no evidence of such metastases.

### MATERIALS AND METHODS

Details of the method, sensitivity and specificity of the radioimmunoassay have been described by Wilde, Orr and Bagshawe (1965, 1967) and by Bagshawe, Wilde and Orr (1966). The method estimates both HCG and luteinising hormone (LH) and requires approximately 1 ml. of the sample fluid. Total CSF protein was estimated by a turbidimetric method using sulphosalicylic acid.

*Samples.*—CSF was obtained by lumbar puncture without preservative or anticoagulant and was stored at −15° C. till assayed. Plasma samples were collected at the time of lumbar puncture with 2 mg./ml. EDTA as anticoagulant.

*Subjects.*—Spinal fluid was obtained from 17 females with post-gestational trophoblastic tumours and from 2 males with choriocarcinoma which probably originated in the testis.

Spinal fluid was also obtained from 10 hospitalised non-pregnant subjects who did not have trophoblastic disease but who underwent lumbar puncture in the course of clinical investigations. Histological evidence of choriocarcinoma was obtained for 10 of the female and for both male patients. Seven female patients were classified as invasive trophoblastic neoplasia (choriocarcinoma or invasive mole) since histological evidence was deficient. There was clinical evidence of intracranial or spinal metastases in 7 instances and autopsy confirmation was obtained in 3 of these.

### RESULTS

The total CSF protein concentrations in the patients studied were either normal or only slightly elevated. Gonadotrophic activity was not found in the CSF of subjects with normal plasma concentrations of luteinising hormone.

The results obtained on patients with elevated plasma gonadotrophins values are shown in Table I.

TABLE I.—*Plasma and CSF HCG Concentrations in Patients with Trophoblastic Tumours*

| Case No. | HCG i.u./ml. Plasma | CSF | Plasma/ CSF ratio | Brain metastases |
|---|---|---|---|---|
| 49 | 0·056 | 0·140 | 0·04 | + |
| 69 | 0·320 | 0·003 | 106 | − |
| 85 | 0·890 | 1·77 | 0·51 | + |
| 86 | 4·00 | 2·00 | 2·0 | + |
| 87 | 51·5 | 1·62 | 32·2 | + |
| 111 | 0·280 | 0·002 | 140 | − |
| 129 | 0·117 | 0·004 | 29·3 | + |
| 132 | 280 | 3·55 | 79·0 | − |
| 133 | 1·52 | 0·009 | 169 | − |
| 136 | 2890 | 550 | 5·26 | + |
| 149 | 17·6 | 0·088 | 200 | − |
| 153 | 18·0 | 0·094 | 192 | − |
| 157 | 12·0 | 0·127 | 94·5 | − |
| 161 | 2·17 | 0·007 | 311 | − |
| 163 | 22·1 | 0·930 | 23·7 | + |
| 164 | 1·88 | 0·009 | 209 | − |
| 169 | 0·700 | 0·006 | 117 | − |
| 191 | 270 | 0·450 | 600 | − |
| 193 | 3·62 | 0·015 | 240 | − |

One pair of estimates of corresponding plasma and CSF concentrations is given for each patient: these were the first estimates for each subject, except in one instance where the estimates given were the first to be obtained after other criteria had confirmed the presence of brain metastases. The HCG concentrations in plasma and CSF are plotted on a double logarithmic scale in Fig. 1 for the 2 groups, those with (●) and those without (△) brain metastases.

For each of the 2 sets of points, the log HCG CSF and log HCG plasma are significantly correlated (group with brain metastases $P < 0{\cdot}02$, group without brain metastases $P < 0{\cdot}001$—two-sided tests) and the data are adequately represented by the simple relationships: CSF HCG i.u./ml. $= a \times$ Plasma HCG i.u./ml. where $a = 0{\cdot}21$ for the group with brain metastases (solid line in Fig. 1) and $a = 0{\cdot}0057$ for the group without brain metastases (dotted line in

FIG. 1.—Relationship between concentration of HCG in plasma and spinal fluid.
● CNS metastases present △ No CNS metastases.

Fig. 1). The value for one subject (B.A.) contributed greatly to the significant level of 0·02 quoted for the brain metastases group but his exclusion did not materially alter the value of " *a* " in the equation of best fit.

### DISCUSSION

The results indicate a close relation between the concentrations of HCG in the plasma and CSF of patients with choriocarcinoma over a wide range of values. Our findings are therefore contrary to those of Zondek (1937), McCormick (1954) and Tashima *et al.*, (1965) who reported that HCG was found in the CSF only when the urinary excretion rate was high and the blood brain barrier overwhelmed by very high titres.

It is suggested that the concept of a threshold effect probably arose from the limitation of the bioassay methods used by earlier workers. Their methods were unsuitable for estimations on plasma and they therefore compared urine excretion rates with CSF concentrations. The renal clearance of HCG is 1·0–1·5 ml./min. so that urine and plasma concentrations tend to be similar. The threshold value reported by these workers was about 200,00 i.u. HCG/day which would correspond to a plasma concentration of about 150–200 i.u. HCG/ml. Since our estimate of the mean plasma : CSF ratio is 177 : 1 the concentration of HCG in the CSF at the "threshold value" would be about 1 i.u. HCG/ml. Although these authors did not give precise estimates of the sensitivity of their methods, it is unlikely that their methods could detect concentrations much lower than 1 i.u. HCG/ml. and their conclusion that HCG was absent from the CSF at lower urinary excretion rates may be attributed to the inability of their methods to detect it.

The radioimmunoassay used in the present study, in common with other biological and immunological methods, does not estimate HCG and LH independently when both are present. The presence of HCG is inferred when the estimates obtained exceed the LH values which are normal for a subject's age, sex and endocrine status. The contribution from LH does not usually exceed the equivalent of 0·2 i.u. HCG/ml. plasma whereas all but 2 of the subjects studied here had higher values. Since all the subjects were known to have trophoblastic tumours which synthesise HCG and since LH was not detected in the CSF of normal, pre-menopausal, non-pregnant subjects by the radioimmunoassay technique, the results have been interpreted only in terms of HCG.

Patients who had intracranial or spinal metastases from choriocarcinoma had higher concentrations of HCG in the CSF relative to the plasma than those who did not, and they were especially high in those patients whose active tumour was located mainly in the brain or spinal cord. Plasma : CSF values which were characteristic of intracranial disease proved to have diagnostic value. For instance, in one patient they gave evidence of an intracranial metastasis some weeks before other objective abnormalities were present. Another patient had evidence of left cerebro-cortical and right orbital metastases at the time of admission and after 6 months' chemotherapy his gonadotrophin excretion had fallen to the normal range. Some weeks after gonadotrophin excretion rates had been persistently in the normal range, but whilst still being treated, it was found that he had a relatively high CSF concentration of HCG. This suggested that viable tumour cells remained in the CNS and following therapy directed at the intracranial tumour, his mean gonadotrophin excretion rate has shown a further fall. Evidence of intracranial growth influences the duration, route and type of therapy so that determination of the CSF HCG concentration is of practical value.

When plasma and CSF concentrations of HCG have been determined their significance can be assessed either by application of the regression line formulae given in the results section or by plotting them on a double logarithmic scale such as is shown in Fig. 1. The ratios of plasma : CSF were above 100 : 1 in most of the patients who did not have intracranial disease and below 35 : 1 in patients who did. It is likely that the amounts of hormone in the plasma and CSF respectively are determined by the anatomical distribution of metastases and their respective endocrine activity so that a wide spread of values is to be expected for patients who have tumours both within and without the CNS. The present evidence is too limited to exclude the possibility that a metastasis may be so located within the CNS that significant amounts of hormone do not reach the CSF directly and a normal plasma : CSF ratio should not be regarded as conclusive evidence of the absence of such a lesion.

It is also important to emphasise that rapid changes in the plasma concentration of HCG are not promptly reflected in the CSF and its relatively low concentration in the CSF in the absence of intracranial metastases, is typical of proteins in general. In comparison with serum, the CSF contains a relatively high proportion of $\beta$-globulin and a low proportion of $\gamma$-globulin (Davson, 1967) but although HCG is a $\beta$-globulin its relative concentrations in the CSF do not appear to be higher than those of proteins in general.

It would therefore be expected that primary changes in the plasma or CSF concentrations would not be promptly reflected in the other compartment. Our preliminary observations on this aspect of the problem suggest that equilibrium may take a week or more to achieve following a rapid thousand-fold change in the plasma concentration. Thus, a CSF sample, obtained soon after a rapid and profound fall in the plasma HCG concentration shows a relatively high concentration of HCG and does not then indicate the presence of CNS metastases. Similarly, a rapidly rising plasma concentration of HCG could temporarily obscure the evidence of an active lesion in the central nervous system. It is therefore essential to take account of recent fluctuations in the plasma concentration and in many instances serial determinations will be necessary.

The detection of intracranial metastases by endocrine studies on the CSF may be applicable to other hormone producing tumours. The observations reported here draw attention to the suitability of radioimmunoassay techniques for the study of specific proteins in CSF.

### SUMMARY

The concentration of chorionic gonadotrophin in the spinal fluid of patients with trophoblastic tumours is directly proportional to its concentration in the plasma. The factor of proportionality is higher in patients with intracranial metastases than in patients without metastases and this fact may provide early evidence of metastases in the brain or spinal cord. The interpretation of plasma: CSF concentrations of the hormone requires recent fluctuations of the plasma concentration to be taken into account.

We wish to thank Dr. M. C. Pike for advice on the statistics. These studies have been supported by grants from the British Empire Cancer Campaign for Research and the Charing Cross Hospital Research Sub-Committee.

### REFERENCES

Aschheim, S. and Zondek, B.—(1927) *Klin. Wschr.*, **6**, 1322.
Bagshawe, K. D., Orr, A. H. and Rushworth, A. G. J.—(1968) *Nature, Lond.*, **217**, 950.
Bagshawe, K. D., Wilde, C. E. and Orr, A. H.—(1966) *Lancet*, i, 1118.
Davson, H.—(1967) 'Physiology of the cerebrospinal fluid'. London (Churchill Ltd).
Ehrhardt, K.—(1931) *Medsche Klin.*, **27**, 426.
Hamburger, C.—(1944) *Acta obstet. gynec scand.*, **24**, 45.
Hirose, T.—(1920) *J. Kinki gynec. Soc.*, **16**.
Mathieu, A.—(1939) *Am. J. Obstet. Gynec.*, **37**, 654.
McCormick, J. B.—(1954) *Obstet. Gynec., N.Y.*, **3**, 58.
Tashima, C. K., Timberger, R., Burdick, R., Leavy, M. and Rawson, R. W.—(1965) *J. clin. Endocr. Metab.*, **25**, 1493.
Vesell, M. and Goldman, S.—(1941) *Am. J. Obstet. Gynec.*, **42**, 272.
Wilde, C. E., Orr, A. H. and Bagshawe, K. D.—(1965) *Nature, Lond.*, **205**, 191.—(1967) *J. Endocr.*, **37**, 23.
Zondek, B.—(1929) *Endokrinologie*, **5**, 429.—(1937) *J. Am. med. Ass.*, **108**, 607.—(1942) *J. Obstet. Gynaec. Br. Commonw.*, **49**, 397.